# Marginal zone lymphoma masquerading as phymatous acne rosacea: a case study

**DOI:** 10.1093/skinhd/vzaf026

**Published:** 2025-04-11

**Authors:** Hina S Baloch, Zhenghao Wang, Joy U L Staniforth, Azaharry Yaakub

**Affiliations:** Department of Dermatology, Norfolk and Norwich University Hospital, Norwich, UK; Department of Dermatology, Norfolk and Norwich University Hospital, Norwich, UK; Norwich Medical School, University of East Anglia, Research Park, Norwich, UK; Department of Histopathology, Addenbrookes Hospital, Cambridge, UK; Department of Dermatology, Norfolk and Norwich University Hospital, Norwich, UK

## Abstract

Marginal zone lymphoma (MZL) is an indolent B-cell lymphoma characterized by considerable heterogeneity in clinical presentation. Cutaneous MZL typically manifests as papules, plaques or nodules, often affecting the trunk and arms. Rare cases of MZL presenting as acne rosacea have been reported; however, these have been primarily reported as granulomatous rosacea. Specific evidence of MZL presenting as phymatous acne rosacea is extremely rare and not well documented in the medical literature. We report a rare case of primary systemic nodal MZL manifesting alongside cutaneous extra-nodal MZL, mimicking rhinophymatous and otophymatous acne rosacea. An 84-year-old White man with a 13-year history of nodal MZL, under active monitoring, presented with erythematous, swollen lesions on the ears and nose. This was initially diagnosed as acne rosacea; however, conventional treatment proved ineffective, and the patient was referred for dermatological evaluation. A skin biopsy from the earlobe revealed a diffuse infiltrate of small lymphoid B cells, positive for CD20, CD79a and BCL2, and negative for CD5 and CD23, consistent with cutaneous MZL. Further imaging revealed systemic involvement, with enlarged lymph nodes above and below the diaphragm and splenomegaly. The patient was started on R-CVP chemotherapy (rituximab, cyclophosphamide, vincristine and prednisolone), leading to significant improvement in both the skin lesions and systemic disease. However, due to chemotherapy intolerance, treatment was discontinued after four cycles. This case highlights a rare presentation of MZL, mimicking the features of phymatous acne rosacea, particularly rhinophyma and otophyma. The resemblance to rosacea, particularly phymatous subtypes, leads to initial misdiagnosis and delays in appropriate treatment. This underlines the importance of considering alternative diagnoses in patients with atypical or nonresponsive dermatological conditions, especially when conventional therapies fail. Early biopsy and histological evaluation are critical for ensuring timely diagnosis and treatment, potentially improving patient outcomes.

## Teaser

Marginal zone lymphoma (MZL) is an indolent B-cell lymphoma characterized by considerable heterogeneity in clinical presentation. In the skin, they typically present as papules, plaques and nodules, commonly appearing on the trunk and arms. Here, we describe the rare case of an 84-year-old male who presented with MZL affecting both earlobes and the nose resulting in redness and swelling, mimicking otophymatous and rhinophymatous acne rosacea.

**What is already known about this topic?**

- We know cutaneous lymphomas have a variety of different presentations, making diagnosis difficult.
- Marginal zone lymphoma (MZL) usually presents on the trunk and limbs, and has mainly been noted to present as granulomatous acne rosacea; however, there is very little literature on cutaneous MZL manifesting as phymatous disease.

**What does this study add?**

- This case illustrates the rare presentation of MZL as phymatous disease, mimicking the presentation of phymatous rosacea, thus adding to currently sparse medical literature.
- This case also stresses the importance of histological investigation in unusual presentations of phymatous diseases and presumed rosacea, especially in new presentations in older patients not responsive to management.

Marginal zone lymphoma (MZL) is a form of non-Hodgkin B-cell lymphoma and is the second most prevalent form of indolent B-cell lymphoma. Recent years have seen a rise in the incidence of MZL overall as well as in its various subtypes, with the incidence of MZL ranging from 0.5 to 2.92 cases per 100 000 person-years, depending on the geographical region.^1^ MZL is classified into three subtypes: mucosa-associated lymphoid tissue (MALT) lymphoma, which accounts for 70% of cases; splenic MZL, representing 20%; and nodal MZL, making up 10%. In some instances, MZL presents with advanced-stage disease where a distinct pattern of organ involvement cannot be identified, and these cases are typically referred to as disseminated MZL.^2^

MZLs may manifest cutaneously as a primary cutaneous malignancy, or as a result of secondary involvement from noncutaneous MZL. While cutaneous MZL most commonly presents as papules, plaques and nodules seen on the arm or trunk, cutaneous B-cell lymphomas have a broad spectrum of clinical presentations, which makes subsequent diagnosis and management challenging.^3,4^

We report a rare case of primary systemic nodal marginal zone lymphoma manifesting alongside cutaneous extra-nodal MZL, mimicking rhinophymatous and otophymatous acne rosacea. This rare case underscores the heterogeneous presentation of MZL, particularly its phymatous appearance, and highlights the importance of a comprehensive physical examination to identify potential systemic features and the necessity of early histological confirmation in all atypical skin presentations.

## Case report

**Figure 1:**
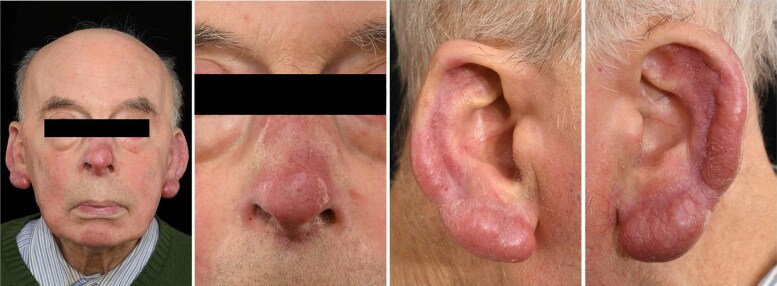
Bilateral erythema and swelling of both earlobes (otophyma) and redness and swelling of the nose (rhinophyma).

An 84-year-old Caucasian man with a 13-year history of nodal marginal zone lymphoma under active monitoring was observed to have swollen erythematous lesions on his ears and nose during a routine haematology follow-up. An initial diagnosis of acne rosacea was made, which proved non-responsive to two courses of topical therapies. Subsequently, he was started on oxytetracycline in the community and referred to our secondary care dermatology clinic. On examination (Figure 1), he exhibited phymatous changes to the nose and both ears, with redness and firm swelling of the earlobes extending to the helix and antihelix bilaterally. There was redness and swelling of the tip of the nose, along with small papules on the right side of the neck. Systemic examination revealed submandibular lymphadenopathy and splenomegaly.

## Investigation

**Figure 2:**
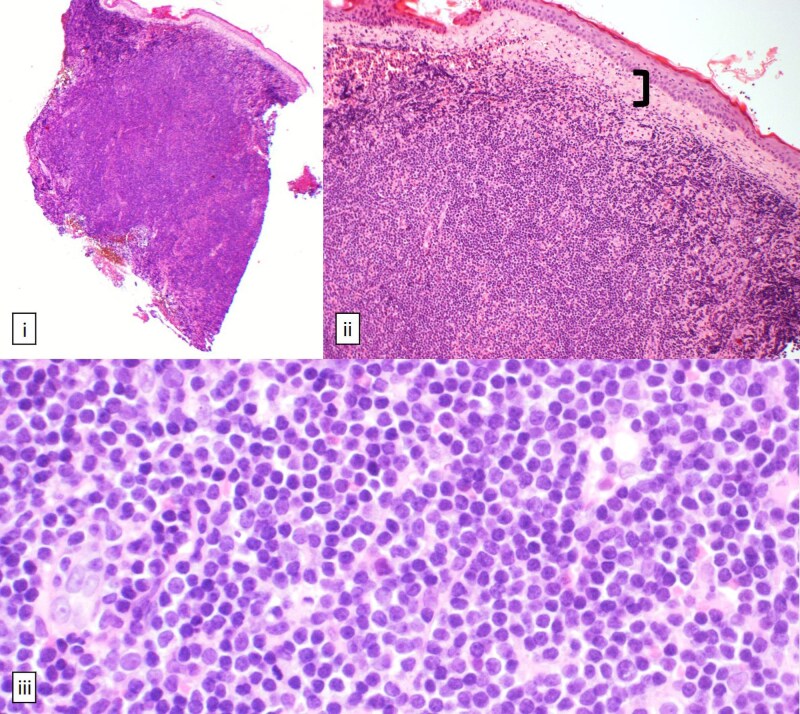
Haematoxylin and eosin staining [(i) 40×, (ii) 100×, (iii) 400×]. (ii) Dermal dense cellular infiltrate separated from epidermis by a grenz zone [(ii), bracket]. (iii) Infiltrate comprises small, monomorphic lymphoid cells showing high nuclear-to-cytoplasmic ratios with rounded nuclei and stippled chromatin.

**Figure 3:**
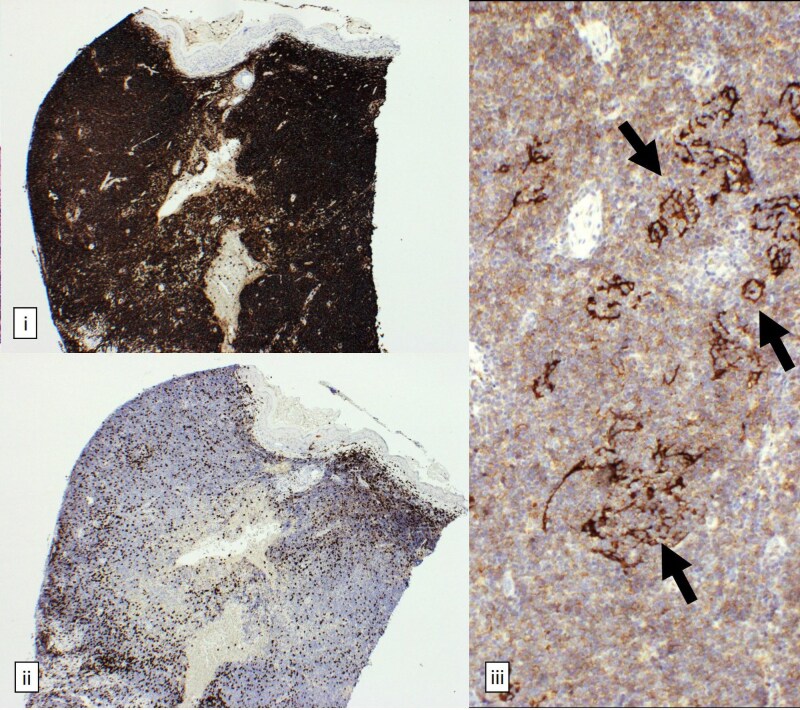
Immunohistochemistry [(i) and (ii) 40×, (iii) 100×]. (i) Infiltrating CD20+ B cells. (ii) Scattered admixed CD3+ T cells and underlying disrupted follicular dendritic cell meshworks [(iii), arrows]. B cells express CD79a and BCL2, weakly express CD21 (iii); and are negative for CD3 (ii).

Given the atypical presentation inconsistent with classical rosacea, skin biopsies were performed on the left earlobe and right side of the neck. Skin biopsy of the right neck demonstrated granulomatous rosacea. However, histological examination of the left earlobe (Figure 2) revealed a diffuse, dense monomorphic infiltrate of dermis-replacing small lymphoid B cells with rounded nuclei and stippled chromatin. Immunohistochemistry (Figure 3) demonstrated positive expression of CD20, CD79a and BCL2, weak expression of CD21, and no expression of CD3, CD5, CD10, BCL6, cyclin D1 and CD30. There were scattered underlying disrupted CD21-positive follicular dendritic cell meshworks, with no morphological evidence of epithelioid malignancy. These features were in keeping with cutaneous MZL.

A computed tomography (CT) scan of the neck, thorax, abdomen and pelvis revealed enlarged lymph nodes both above and below the diaphragm, along with splenomegaly, demonstrating diffuse systemic lymphoma.

## Treatment and follow-up

**Figure 4:**
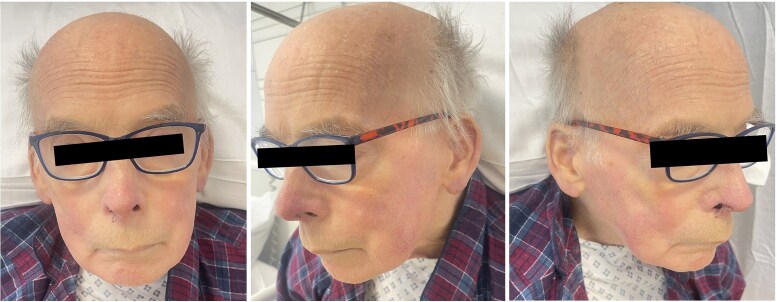
Resolution post-chemotherapy.

Considering his disseminated MZL, he was started on R-CVP chemotherapy (rituximab, cyclophosphamide, vincristine and prednisolone). After completing four cycles of chemotherapy, his skin condition showed dramatic improvement (Figure 4). Unfortunately, he was unable to tolerate further chemotherapy and now remains under active monitoring. Subsequent CT scans have shown subcentimetre lymph nodes and a reduction in spleen size.

## Discussion

Phymas, encompassing conditions such as rhinophyma and otophyma, represent an uncommon but significant manifestation of rosacea and illustrate the complex spectrum of acne rosacea presentation. Phymas develop in approximately 5–10% of those affected by acne rosacea, characterized by sebaceous hyperplasia, fibrosis and localized lymphoedema. This commonly affects the nose (rhinophyma), but may also affect the chin (gnathophyma), forehead (metophyma) and ears (otophyma). Cutaneous presentations of MZL include a broad range of clinical manifestations, most commonly nodules, plaques or papules often affecting the trunk or limbs. While there have been rare cases reported of MZL presenting as acne rosacea,^3–5^ these have been reported as primarily granulomatous rosacea, and evidence of MZL presenting specifically as phymatous disease is extremely sparse and not well documented in the medical literature.

This case is notable due to the rare involvement of both the nose and ears, mimicking rhinophyma and otophyma, highlighting the diverse presentation of MZL and emphasizing the importance of a comprehensive physical examination. Conventional therapies for acne rosacea proved ineffective, and referral to secondary care dermatology and skin biopsy was essential for a definitive diagnosis. Atypical phymatous presentations of disease often lead to misdiagnosis, resulting in mismanagement and delays in appropriate treatment. This highlights the critical need for dermatological expertise and prompt histopathological evaluation to differentiate phymas from mimicking conditions such as inflammatory disorders, lymphomas, cryoglobulinaemia and cryofibrinogenaemia, granulomatous disorders such as sarcoidosis, and squamous cell and basal cell carcinomas. While surgical intervention is often required for advanced cases of phymatous rosacea, recognition of the cutaneous MZL allowed early treatment with R-CVP chemotherapy, resulting in a dramatic improvement in the patient’s skin condition and overall systemic disease.

Prognosis of MZL varies significantly depending on the subtype, with MALT lymphomas exhibiting better outcomes than nodal or splenic variants.^6^ In this case, prompt initiation of chemotherapy led to a marked improvement in cutaneous and systemic symptoms. Nevertheless, the involvement of multiple organ systems, including the skin and lymph nodes, necessitates careful monitoring for disease progression and transformation into more aggressive subtypes, such as diffuse large B-cell lymphoma.^7^ Although rare, lymphoma should be considered in the differential diagnosis of all atypical or nonresponsive rosacea. Early histopathological evaluation is crucial to differentiate between benign and malignant conditions.

The rare phymatous presentation of MZL in this report illustrates the need for dermatologists and clinicians to consider lymphoma in the differential diagnosis of persistent, treatment-resistant rosacea-like conditions, and highlights the importance of histological evaluation in patients with persistent or atypical dermatological findings. Although rosacea, especially phymatous forms, can present similarly, clinicians must maintain a high index of suspicion for underlying malignancies in new cases that do not respond to standard therapy. Early biopsy and histopathology are essential to ensure timely diagnosis and treatment.

## Additional statements

H.S.B. and Z.W., as co-first authors, contributed equally.

## Data Availability

There are no new data associated with this article.
